# Lower-Income Countries That Face The Most Rapid Shift In Noncommunicable Disease Burden Are Also The Least Prepared

**DOI:** 10.1377/hlthaff.2017.0708

**Published:** 2017-11-01

**Authors:** Thomas J. Bollyky, Tara Templin, Matthew Cohen, Joseph L. Dieleman

**Affiliations:** 1 Senior fellow for global health, economics, and development at the Council on Foreign Relations, in Washington, D.C; 2 Graduate student in the Department of Health Research and Policy, Stanford University, in Palo Alto, California; 3 Research associate for global health, economics, and development at the Council on Foreign Relations; 4 Assistant professor at the Institute for Health Metrics and Evaluation, University of Washington, in Seattle

## Abstract

Demographic and epidemiological changes are shifting the disease burden from communicable to noncommunicable diseases in lower-income countries. Within a generation, the share of disease burden attributed to noncommunicable diseases in some poor countries will exceed 80 percent, rivaling that of rich countries, but this burden is likely to affect much younger people in poorer countries. The health systems of lower-income countries are unprepared for this change. We examined the shift to noncommunicable diseases and estimated preparedness for the shift by ranking 172 nations using a health system capacity index for noncommunicable disease. We project that the countries with the greatest increases in the share of disease burden attributable to noncommunicable disease over the next twenty-five years will also be the least prepared for the change, as they ranked low on our capacity index and are expected to have the smallest increases in national health spending. National governments and donors must invest more in preparing the health systems of lower-income countries for the dramatic shift to noncommunicable diseases and in reducing modifiable noncommunicable disease risks.

The combination of demographic and epidemiological changes is producing rapid shifts in the disease profile of many low-income nations. Cancers, diabetes, cardiovascular diseases, chronic respiratory illnesses, and other noncommunicable diseases are on the rise in low-income countries because of the increased prevalence of key modifiable behavioral risks, such as unhealthy diets and tobacco use, and reductions in the infectious diseases that disproportionately kill children and adolescents. At the same time, population aging and growth are amplifying the speed of the shift from communicable, maternal, neonatal, and nutritional diseases to noncommunicable diseases.^1^ Many poorer countries such as Bangladesh and Botswana are experiencing major reductions in fertility. As a consequence, between 1980 and 2015 the median age in these countries increased from seventeen to twenty-six years and from seventeen to twenty-four years, respectively.^2^ As the number and share of adults continue to grow in poor countries, so will the burden of the noncommunicable diseases that disproportionately affect them.

The expected pace and scale of the shift in disease profile to noncommunicable diseases will present significant challenges to the health care systems of many lower-income nations.^3^ Many of these countries have historically focused on acute care for patients with infectious, maternal, and neonatal diseases, rather than on the preventive or chronic care that many noncommunicable diseases require.^4,5^ Government health spending remains low in poor nations. The average government of a low-income country spends $23 per person annually on health (purchasing power parity adjusted).^6^ In comparison, the US government spends $3,860 per person on health, while the UK government spends $2,695.^7^ In many low and lower-middle-income countries, most health services and medicines are still purchased out of pocket or by donors. In 2014, 29.1 percent of health spending was financed out of pocket, while 35.7 percent was financed by donors.^6^ When prepaid financing is not available through insurance or via donors, many important health care services may be too expensive for poor households.^8^

With little access to preventive and primary care, working-age people in lower-income nations are more likely to develop and receive late diagnoses for breast and cervical cancer, hypertension, and other noncommunicable diseases.^9,10^ Without access to chronic care and with limited resources to pay for medical treatment out of pocket, working-age people in these lower-income countries are more likely to become disabled or die young.

It has long been observed that lower-income nations are experiencing an epidemiological transition toward noncommunicable diseases and that the health systems of many of these countries are not adequately prepared for that shift.^11–16^ Understanding the expected speed and scale of the shift to noncommunicable diseases at the country level and using externally validated data to estimate the preparedness of each country’s health system for that shift is important for the health planning, budgeting, and policy formulation of national governments. It is also important for mobilizing more international donor support for noncommunicable diseases, which receive only 2 percent of overseas development assistance for health worldwide.^17^

We measured the expected speed and scale of the epidemiological shift to noncommunicable diseases for 172 countries and the relative preparedness of their national health systems for that change. We focused on three interconnected pieces of evidence. First, we calculated the shift to noncommunicable diseases that occurred in each country from 1990 to 2015 and projected the additional shift that can be expected to occur by 2040. Second, we connected these projections to expected per capita increases in health spending for each country. Finally, we constructed the first health system capacity index for noncommunicable diseases, adapted from health system building blocks of the World Health Organization (WHO), and we compared noncommunicable disease preparedness and expected rates of health burden.

## Study Data And Methods

Population And Aging Data And Projections Demographic data were extracted from the UN World Population Prospects database.^2^ These data track and predict age-specific populations for each country in the world through 2100. For the population forecast, we used the medium variant, which assumes that nations will undergo a demographic transition—with child mortality and total fertility declining and population growth and aging increasing—at rates similar to the average country that has undergone such a transition in the past.

**health burden data and projections** Epidemiological data were collected from the Global Burden of Disease 2015 study.^1,18^ We extracted age-, sex-, and cause-specific disability-adjusted life-year (health burden) estimates for 315 causes of disease and disability for 195 countries in the period 1990–2015. We aggregated these data into three categories: communicable, maternal, neonatal, and nutritional diseases; noncommunicable diseases; and injuries. For a list of the noncommunicable diseases included in these data, see Table 3.3 in the online Appendix.^19^

To create simple and transparent mortality and health burden projections, we calculated the annualized rate of change from 2005 to 2015 of health burden for the three disease categories listed above for each country, and for each sex-specific five-year age group. We used the observed annualized rate of change calculated from the data for 2005–15 to project health burden rates for each country and age and sex group through 2040. We estimated total health burden by multiplying the World Population Prospects’ projected populations by the projected health burden rates.

To confirm the robustness of these projections, we conducted a sensitivity analysis using an alternative model that forecast all causes of health burden except HIV/AIDS using all years of data. In addition, we assessed two other alternative scenarios, one in which population characteristics remained constant and one in which noncommunicable disease death rates remained constant. The results of these additional analyses are in Figures 2.2.1–13 in the Appendix.^19^

To illustrate the relative impact of changes in population size, population aging, and changes in prevalence of noncommunicable diseases, we split the increases in disability-adjusted life-years using demographic decomposition.^20^ This algebraic method calculated the differences in standardized rates over time to measure the relative impact of each of the three factors.

**health spending data and projections** Health spending data were obtained from the Institute for Health Metrics and Evaluation’s Financing Global Health 2016 database.^6,7^ Data and projections are available for 184 countries for the period 1995–2040.

**noncommunicable disease health system capacity index** The WHO has specified six health system building blocks:^21^ health service delivery, health workforce, health information systems, access to essential medicines, health financing, and leadership and governance. We constructed a health system capacity index for noncommunicable disease based on the four building blocks for which there are externally validated data, as explained below. Previous studies have also constructed indices reflecting the WHO building blocks, but we found none that specifically target noncommunicable diseases.^22,23^

Indicators included in our index are hospital beds per 1,000 population and number of surgeries per 100,000 population (WHO building block: health service delivery), physicians per 1,000 population and the percentage of births with skilled attendants (WHO building block: health workforce), total health expenditure as a percentage of gross domestic product (WHO building block: health financing), and a variable identifying the degree to which each country has implemented tobacco control policies (WHO building block: leadership and governance). The remaining two building blocks (health information systems and access to essential medicines) were not included because externally validated data for a sufficient number of countries were not available. The WHO has done some work to collect information on noncommunicable disease preparedness indicators for these two categories, but external validation of these data has not yet been completed.^24–26^

The indicators in our index correspond to demands that noncommunicable diseases impose on national health systems. Most such diseases are chronic, require health delivery infrastructure and skilled workforces, and are costly to treat.^27^ For example, a recent study in Tanzania revealed that noncommunicable diseases were responsible for only 27 percent of the country’s health burden but for nearly half of its hospital admissions and hospitalization days.^28^ Studies conducted in the United States and New Zealand showed higher rates of inpatient surgical procedures for the treatment of noncommunicable diseases than for that of communicable, maternal, and neonatal diseases (34 percent versus 24 percent in the United States and 32 percent versus 16 percent in New Zealand).^29,30^ Late diagnosis of many cases of cancer, diabetes, and cardiovascular disease in low-income nations increases the demand for inpatient surgical procedures. Our index includes indicators for the numbers of hospital beds, surgeries, and physicians, as well as the use of birth attendants, as a rough proxy for the overall availability of skilled health workers. The health care financing required to prevent, diagnose, and treat noncommunicable diseases is shown in the indicator for health expenditure, measured by its percentage of gross domestic product.

We used principal components analysis of the available data to generate a health system capacity index for 172 countries in 2014. As a sensitivity analysis, we completed the principal components analysis of a subset of countries, using the nonvalidated indicator of whether the country had reported to the WHO that it had a multisectoral, national, and integrated strategy to manage and prevent noncommunicable diseases and risk factors. Results of this sensitivity analysis, which are reported in part 2.1 of the Appendix,^19^ were qualitatively the same as those using all 172 countries and show that this additional variable has little impact on the index. A further exploration of the index’s characteristics is in Table 1.2.1 in the Appendix.^19^

All analyses were completed using Stata, version 14.1, and R, version 3.3.2.

**limitations** Our study had several limitations. First, all data considered for this analysis are national averages, which potentially mask inequalities and subnational spatial patterns within a country.^31–33^ Although many policy decisions are made at the national level and comparisons at this level are quite common, using national averages hides the variation within countries and may incorrectly characterize some individuals.

Second, forecasting population change, health burden, and health care spending is based on past trends and assumptions about the future, which are not certain to be accurate. Even population projections, which are the easiest of the three to undertake, are still methodologically challenging, require some assumptions, and can be based on a diverse set of methods that can lead to a diverse set of forecasts.^34–36^ We used changes from 2005 to 2015 in age-specific health burden to project burden rates to 2040. Using all available Global Burden of Disease data (for 1990–2015) instead would have inappropriately exaggerated the expected future HIV burden, which peaked in 2004–05.^37^ These methods were tested using an alternative time period, with results presented in Appendix Part 2.2.^19^ The modeling technique accounted for population growth, aging, and other expected demographic changes in each country, but otherwise it made predictions on the basis of limited information. We believe that this methodology produced reliable country-level estimates of future noncommunicable disease burden based on recent trends, but its use should be replicated as more detailed and sophisticated forecasts of future health burden become available.

Third, high-quality data on disease burden in the poorest countries are limited, especially with regard to noncommunicable diseases. To mitigate this limitation, the Global Burden of Disease Study draws upon data and expertise from more than 2,500 collaborators in 133 countries and uses statistical methods to correct for bias and missing data.

Finally, there is a dearth of comparable and validated data that focus specifically on national health systems’ capacity to prevent and treat noncommunicable diseases. Our methodology and resulting index assumed that countries with relatively greater financial investment in health, hospital infrastructure, tobacco control, physicians, and skilled health workers were better equipped to deal noncommunicable diseases, compared to other countries. We believe that the reasoning behind this assumption is sound, but future assessments of national health system capacity would be improved with the use of more regularly reported and validated data on access to essential medicines for noncommunicable diseases, the provision of noncommunicable disease–specific health services, and control of important modifiable health risks other than tobacco use.

## Study Results

Our study had four main results. First, our analysis showed that low- and lower-middle-income nations are expected to see dramatic increases in the burden of premature death and disability from noncommunicable diseases by 2040 (Exhibit 1). In most geographic regions, these increases will result from large demographic changes (population aging and growth) that will not be sufficiently offset by the much more modest improvement in the rates of morbidity and mortality of noncommunicable diseases (Exhibit 2).^38^ In lower-middle-income nations, most of the increase in noncommunicable diseases will be experienced in populations ages thirty-five and older (Exhibit 1). In low-income nations, the surge in disability-adjusted life-years (health burden) lost to noncommunicable diseases will be experienced across all age groups, but particularly in working-age adults (people ages 25–64) and infants. In contrast, increases in the toll of noncommunicable diseases in upper-middle- and high-income nations will be in older populations, with declines occurring in many younger-age cohorts (Appendix Figure 1.2.1).^19^

**EXHIBIT 1:**
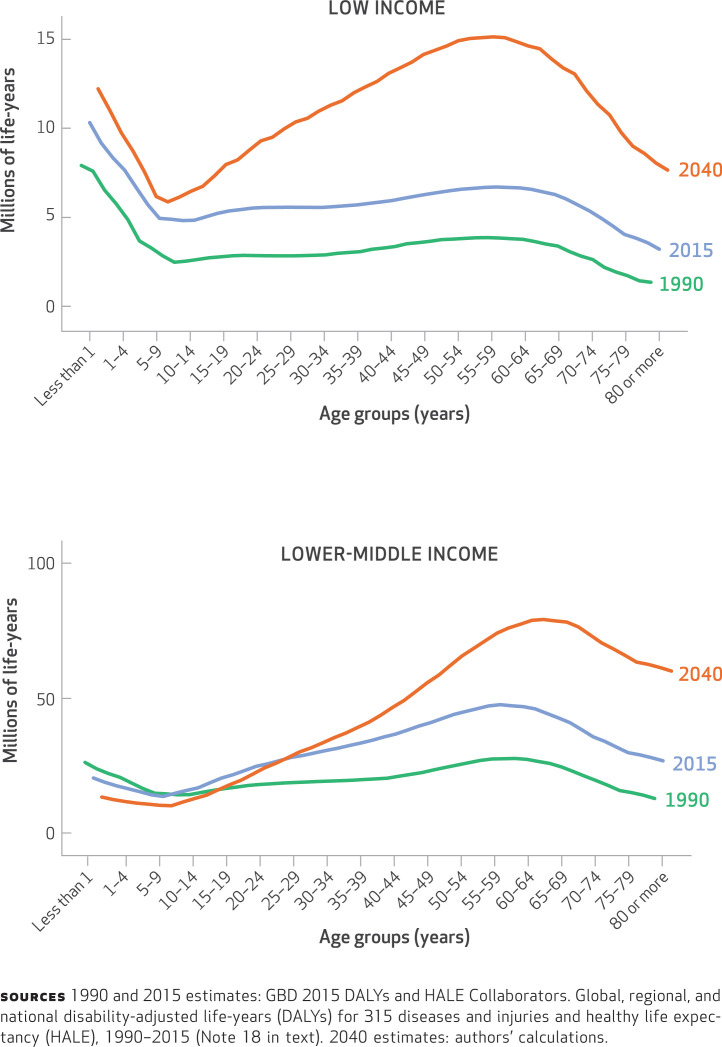
Millions of disability-adjusted life-years lost due to noncommunicable diseases, by age group and country income category, 1990, 2015, and 2040

**EXHIBIT 2:**
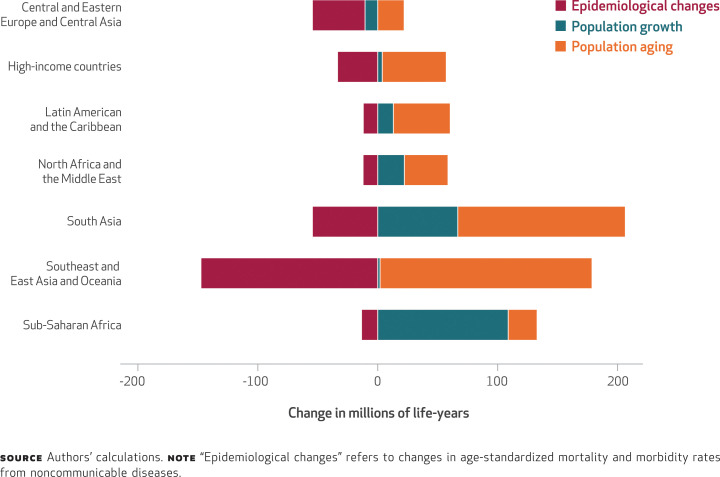
Changes from 2015 to 2040 in millions of disability-adjusted life-years lost due to noncommunicable diseases, by geographic region or country income category and driver

Second, our analysis showed that the share of health burden that arises from noncommunicable diseases will increase significantly in many low- and lower-middle-income nations by 2040 (Exhibit 3). In particular, countries such as Bangladesh, Myanmar, Iran, the Philippines, and Vietnam will have gone from having a health burden dominated by communicable, maternal, neonatal, and nutritional diseases to having noncommunicable diseases represent more than four-fifths of their health burden within a generation or two. At that point, the noncommunicable disease burden in these lower-income countries will be roughly the same as in the United States and other wealthy nations (Exhibit 3), but the burden will occur in much younger people in the poorer countries (Appendix Figure 1.2.1).^19^

**EXHIBIT 3:**
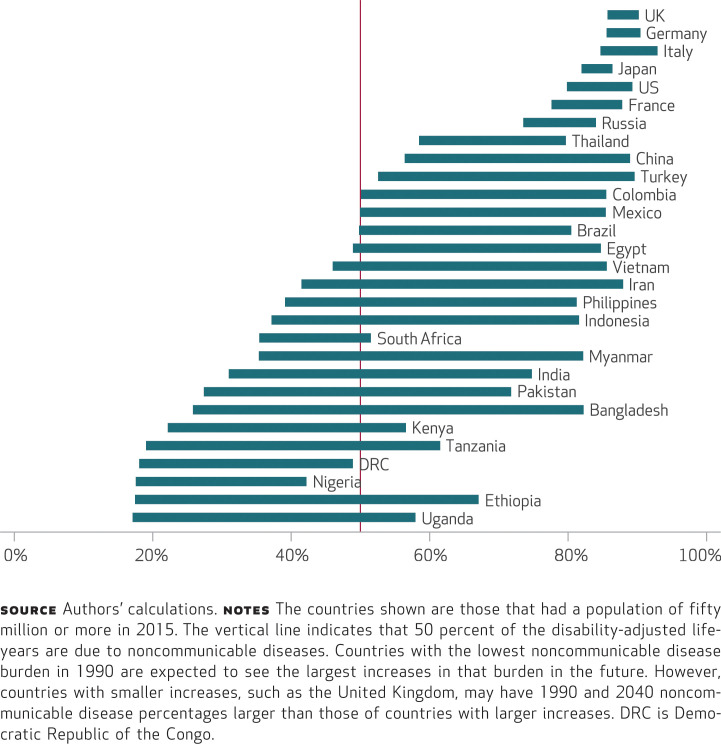
Changes from 1990 to 2040 in percentages of disability-adjusted life-years lost due to noncommunicable diseases in populous countries

Third, our analysis showed that the countries that will experience the greatest increases in death and disability from noncommunicable diseases over the next twenty-five years are also projected to have the smallest increases in health spending (Exhibit 4). Here, too, there is a significant difference across income groups. High-income countries are projected to have relatively modest increases in the percentages of health burden due to noncommunicable diseases and significant increases in their resources to address those health needs. The one exception is China, which is not yet a high-income nation but which has slowed its increase in the percentage of health burden due to noncommunicable diseases and is expected to significantly expand its total health spending.

**EXHIBIT 4:**
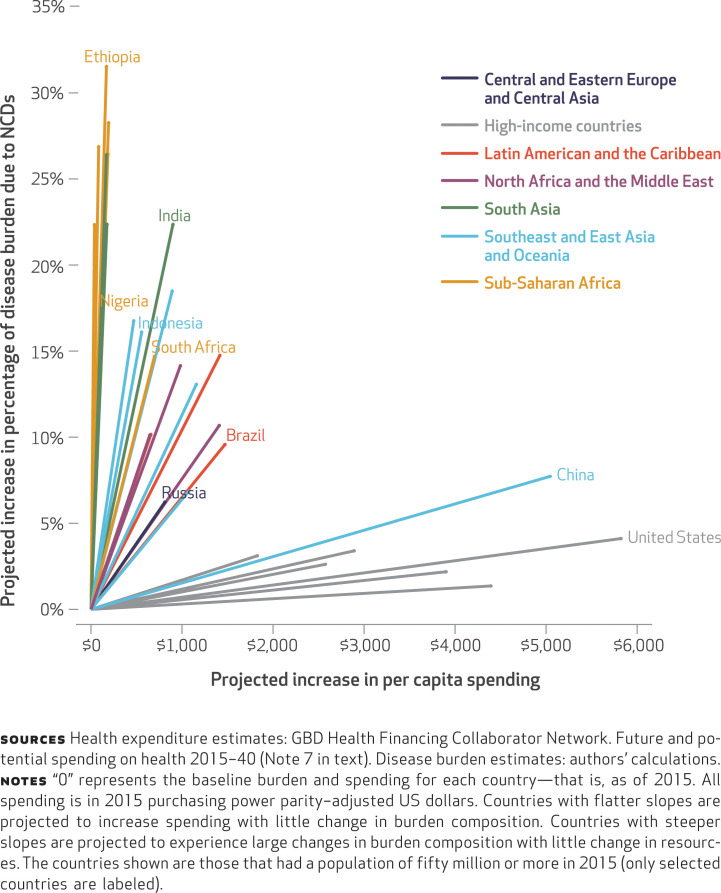
Projected increases in total health spending per capita and in percentage of health burden due to noncommunicable diseases (NCDs) from 2015 to 2040, by geographic region or country income category

Fourth, our analysis showed that countries that are projected to have the greatest increase in their noncommunicable disease burden as a share of health burden are also ranked lowest (least prepared) in our health system capacity index for noncommunicable diseases (Exhibit 5). African nations do particularly poorly on this measure, but the health systems of a handful of countries (such as Bangladesh and India) from other regions are also shown to be unprepared for the shift in their disease profiles to noncommunicable diseases.

**EXHIBIT 5:**
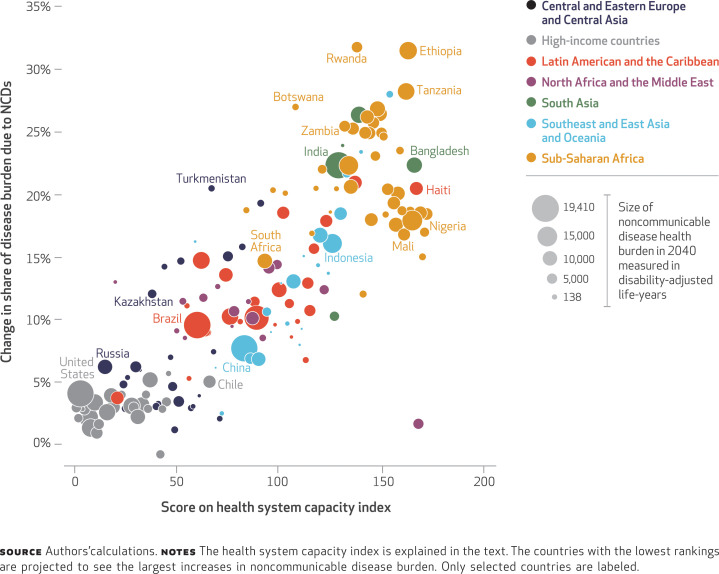
Projected change from 2015 to 2040 in percentage of disease burden due to noncommunicable diseases (NCDs), by score on the health system capacity index

## Discussion

This research shows that the burden of premature noncommunicable diseases is growing quickly in lower-income countries. This growth is fastest in countries with little forecasted change in health spending and with health systems that are not well equipped to manage and treat noncommunicable diseases. We draw three conclusions from our analysis.

First, there is a clear need for increased investment in reducing key modifiable risk factors to reduce the speed and scale of the shift to noncommunicable diseases in lower-income nations. In a previous study published in Health Affairs, we showed that countries that have achieved significant reductions in their premature noncommunicable disease burden have also reduced the prevalence of tobacco use and household air pollution.^39^ There are also significant links between reductions in premature noncommunicable disease mortality and rates of obesity, air pollution, and binge drinking, but few of the countries that have achieved reductions in premature noncommunicable disease mortality managed to lower those risks.^39^ Even with reductions in the risks, a robust and cost-effective health system will remain important for responding to the staggeringly fast rise in premature death and disability from noncommunicable diseases in poorer nations.

Second, more investment is needed in lower-cost ways to elevate primary care as the main platform for responding to noncommunicable diseases in the health systems of low-income nations.^40^ The World Health Organization and the World Economic Forum have recommended packages of cost-effective “best buy” clinical services that can be provided in primary care.^41^ These interventions include educating patients about the risks of unhealthy diets and physical inactivity and about low-cost primary and secondary prevention measures for heart attacks and strokes. However, primary care in most low- and lower-middle-income countries is focused on episodic care and is poorly situated to deliver the access to affordable prevention, diagnosis, and treatment services that many noncommunicable diseases require. There is evidence that increased use of lower-cost community health workers can increase the coverage and quality of care provided by overburdened health systems, especially in rural settings.^5,42,43^ In the HIV/AIDS context, the practice of shifting care to lower-skilled nurses and health workers, known as task shifting, has been used effectively to compensate for a severely limited workforce.^44–46^ These strategies may prove useful in the noncommunicable disease context as well.^47^

Third, lower-income nations must increase the resources that they devote to their health systems. Even with innovative and cost-effective approaches to service delivery, the health systems of these lower-income nations will require more resources to adapt to the expected speed and scale of the transition to noncommunicable diseases. Many of these nations that will experience the greatest increase in death and disability from noncommunicable diseases spend less on health than one would expect based on their income and historical trends. Ghana, Indonesia, Laos, and Nigeria are examples of countries that are expected to have large increases in noncommunicable disease health burden as a percentage of total burden, but they spend less than would be expected, even after including support in the form of aid from external donors.^48^

Countries’ failure to increase health spending can have consequences that extend well beyond the higher rates of unnecessary death and disability. At the household level, premature death and disability from noncommunicable diseases often result in less income and more catastrophic health expenditures.^22,49–51^ Adults who have grown up amid widespread poverty and deprivation may be more prone to functional declines from noncommunicable diseases at younger ages.^52^ Given the scale of the rise of noncommunicable diseases, failing to respond to this shift in the disease profile will mean higher health and welfare expenditures for countries and may reduce national productivity and competitiveness.^53,54^

## Policy Implications

Donor-funded global health programs must evaluate how to spur improved access to critical noncommunicable disease prevention and treatment in lower-income nations that are experiencing rapid demographic and epidemiological shifts. In these countries, the median age of the population is increasing, and much of the health burden is already attributable to noncommunicable diseases.^55^ However, development assistance for health in these nations continues to be earmarked for HIV/AIDS (25 percent) and maternal and childhood health (29 percent).^3,48^ Lower-income countries such as Bangladesh, Ethiopia, and Indonesia are expected to continue to make major reductions in mortality from infectious and childhood diseases, with the support of international donors. As people with HIV/AIDS live longer as a result of greater access to antiretroviral treatment, they are more likely to develop noncommunicable diseases due to aging and HIV-related comorbidities.^56^ Donor investments in HIV/AIDS and other infectious diseases should continue, and the many contributions they have made to reducing the incidence and mortality of HIV/AIDS should be celebrated. However, it is not sustainable to devote significant resources to fighting treatable and preventable communicable diseases only to watch the same patient populations prematurely succumb to equally treatable and preventable noncommunicable diseases. The greater integration of primary and secondary prevention programs into global heath platforms to address HIV/AIDS has worked in other contexts and should be expanded in the context of noncommunicable diseases.^57–59^

More research is needed to assess how to leverage other global health platforms to implement low-cost prevention and control strategies for noncommunicable diseases as well. Shifting donor programs from disease-focused goals to more outcome-oriented measures and investing in the collection of data to monitor those measures would increase the accountability and efficiency of global health investments and help transition important health programs to local country ownership.^60^ Recent reports from the Council on Foreign Relations and the National Academies of Sciences, Engineering, and Medicine (US) likewise urge adopting cardiovascular health and cancer prevention as top priorities for future US investment in global health.^3,61^

## Conclusion

The recent launch of Resolve to Save Lives, a philanthropy-funded $225 million initiative that will seek to improve hypertension control in lower-income countries, provides hope for more donor interest in the rising tide of noncommunicable disease in these nations.^62^ This research shows that the time for donors and lower-income country governments to increase their investment in preventing and treating noncommunicable diseases is now.

Funding was provided by Bloomberg Philanthropies and the Bill & Melinda Gates Foundation. This is an open access article distributed in accordance with the terms of the Creative Commons Attribution (CC BY 4.0) license, which permits others to distribute, remix, adapt and build upon this work, for commercial use, provided the original work is properly cited. See: https://creativecommons.org/licenses/by/4.0/

## NOTES

1 GBD 2015 Mortality and Causes of Death Collaborators. Global, regional, and national life expectancy, all-cause mortality, and cause-specific mortality for 249 causes of death, 1980–2015: a systematic analysis for the Global Burden of Disease Study 2015. Lancet. 2016; 388(10053):1459–544.

2 United Nations. World population prospects: the 2015 revision, methodology of the United Nations population estimates and projections [Internet]. New York (NY): United Nations; 2015 [cited 2017 Sep 25]. (Working Paper No. ESA/P/WP.242). Available from: https://esa.un.org/unpd/wpp/publications/Files/WPP2015_Methodology.pdf

3 Council on Foreign Relations. The emerging global health crisis: noncommunicable diseases in low- and middle-income countries [Internet]. New York (NY): CFR; c 2014 [cited 2017 Sep 25]. (Independent Task Force Report No. 72). Available from: https://www.cfr.org/sites/default/files/report_pdf/TFR72_NCDs.pdf

4 Reidpath DD, Allotey P. The burden is great and the money little: changing chronic disease management in low- and middle-income countries. J Glob Health. 2012;2(2): 020301.

5 Checkley W, Ghannem H, Irazola V, Kimaiyo S, Levitt NS, Miranda JJ, et al. Management of NCD in low- and middle-income countries. Glob Heart. 2014;9(4):431–43.

6 Global Burden of Disease Health Financing Collaborator Network. Evolution and patterns of global health financing 1995–2014: development assistance for health, and government, prepaid private, and out-of-pocket health spending in 184 countries. Lancet. 2017;389(10083): 1981–2004.

7 Global Burden of Disease Health Financing Collaborator Network. Future and potential spending on health 2015–40: development assistance for health, and government, prepaid private, and out-of-pocket health spending in 184 countries. Lancet. 2017;389(10083):2005–30.

8 PwC. From vision to decision: Pharma 2020 [Internet]. London: PwC; [cited 2017 Sep 25]. Available from: https://www.pwc.com/gx/en/pharma-life-sciences/pharma2020/assets/pwc-pharma-success-strategies.pdf

9 Knaul FM, Bhadelia A, Gralow J, Arreola-Ornelas H, Langer A, Frenk J. Meeting the emerging challenge of breast and cervical cancer in low- and middle-income countries. Int J Gynaecol Obstet. 2012;119(Suppl 1): S85–8.

10 Ibrahim MM, Damasceno A. Hypertension in developing countries. Lancet. 2012;380(9841):611–9.

11 Murray CJL, Lopez editors. AD The global burden of disease. Cambridge (MA): Harvard University Press; 1996.

16 Nyaaba GN, Stronks K, de-Graft Aikins A, Kengne AP, Agyemang C. Tracing Africa’s progress towards implementing the Non-Communicable Diseases Global Action Plan 2013–2020: a synthesis of WHO country profile reports. BMC Public Health. 2017;17(1):297.

17 Dieleman JL, Yamey G, Johnson EK, Graves CM, Haakenstad A, Meara JG. Tracking global expenditures on surgery: gaps in knowledge hinder progress. Lancet Glob Health. 2015; 3(Suppl 2):S2–4.

19 To access the Appendix, click on the Details tab of the article online.

20 Das Gupta P. Standardization and decomposition of rates: a user’s manual [Internet]. Washington (DC): Bureau of the Census; 1993 Oct [cited 2017 Sep 25]. (Current Population Report No. P23-186). Available from: https://www.census.gov/content/dam/Census/library/publications/1993/demo/p23-186.pdf

21 World Health Organization. Monitoring the building blocks of health systems: a handbook of indicators and their measurement strategies [Internet]. Geneva: WHO; c 2010 [cited 2017 Sep 25]. Available from: http://www.who.int/healthinfo/systems/WHO_MBHSS_2010_full_web.pdf

22 Engelgau MM, El-Saharty S, Kudesia P, Rajan V, Rosenhouse S, Okamoto K. Capitalizing on the demographic transition: tackling noncommunicable diseases in South Asia. Washington (DC): World Bank; 2011 Jun.

23 Obermann K, Chanturidze T, Richardson E, Tanirbergenov S, Shoranov M, Nurgozhaev A. Data for development in health: a case study and monitoring framework from Kazakhstan. BMJ Glob Health. 2016; 1(1):e000003.

24 World Health Organization. Assessing national capacity for the prevention and control of noncommunicable diseases: global survey [Internet]. Geneva: WHO; c 2016 [cited 2017 Sep 20]. Available from: http://apps.who.int/iris/bitstream/10665/246223/1/9789241565363eng.pdf

26 Samb B, Desai N, Nishtar S, Mendis S, Bekedam H, Wright A, et al. Prevention and management of chronic disease: a litmus test for health-systems strengthening in low-income and middle-income countries. Lancet. 2010;376(9754):1785–97.

27 Muka T, Imo D, Jaspers L, Colpani V, Chaker L, van der Lee SJ, et al. The global impact of non-communicable diseases on healthcare spending and national income: a systematic review. Eur J Epidemiol. 2015;30(4): 251–77.

28 Peck RN, Green E, Mtabaji J, Majinge C, Smart LR, Downs JA, et al. Hypertension-related diseases as a common cause of hospital mortality in Tanzania: a 3-year prospective study. J Hypertens. 2013; 31(9):1806–11.

29 Rose J, Weiser TG, Hider P, Wilson L, Gruen RL, Bickler SW. Estimated need for surgery worldwide based on prevalence of diseases: a modelling strategy for the WHO Global Health Estimate. Lancet Glob Health. 2015; 3(Suppl 2):S13–20.

30 Rose J, Chang DC, Weiser TG, Kassebaum NJ, Bickler SW. The role of surgery in global health: analysis of United States inpatient procedure frequency by condition using the Global Burden of Disease 2010 framework. PLoS One. 2014;9(2): e89693.

31 Pannarunothai S, Mills A. The poor pay more: health-related inequality in Thailand. Soc Sci Med. 1997; 44(12):1781–90.

33 Gething PW, Casey DC, Weiss DJ, Bisanzio D, Bhatt S, Cameron E, et al. Mapping Plasmodium falciparum mortality in Africa between 1990 and 2015. N Engl J Med. 2016; 375(25):2435–45.

34 Raftery AE, Li N, Ševčíková H, Gerland P, Heilig GK. Bayesian probabilistic population projections for all countries. Proc Natl Acad Sci U S A. 2012;109(35):13915–21.

36 Keilman N. How accurate are the United Nations World Population Projections? Popul Dev Rev. 1998; 24(Suppl):15–41.

37 GBD 2015 HIV Collaborators, Wang H, Wolock TM, Carter A, Nguyen G, Kyu HH, et al. Estimates of global, regional, and national incidence, prevalence, and mortality of HIV, 1980–2015: the Global Burden of Disease Study 2015. Lancet HIV. 2016;3(8):e361–87.

38 Roth GA, Forouzanfar MH, Moran AE, Barber R, Nguyen G, Feigin VL, et al. Demographic and epidemiologic drivers of global cardiovascular mortality. N Engl J Med. 2015; 372(14):1333–41.

39 Bollyky TJ, Templin T, Andridge C, Dieleman JL. Understanding the relationships between noncommunicable diseases, unhealthy lifestyles, and country wealth. Health Aff (Millwood). 2015;34(9):1464–71.

40 Kruk ME, Nigenda G, Knaul FM. Redesigning primary care to tackle the global epidemic of noncommunicable disease. Am J Public Health. 2015;105(3):431–7.

41 World Economic Forum. From burden to “best buys”: reducing the economic impact of non-communicable diseases in low- and middle-income countries [Internet]. Geneva: The Forum; c 2011 [cited 2017 Sep 26]. Available from: http://apps.who.int/medicinedocs/documents/s18804en/s18804en.pdf

42 Farzadfar F, Murray CJ, Gakidou E, Bossert T, Namdaritabar H, Alikhani S, et al. Effectiveness of diabetes and hypertension management by rural primary health-care workers (Behvarz workers) in Iran: a nationally representative observational study. Lancet. 2012;379(9810): 47–54.

43 Habibzadeh F. The control of noncommunicable diseases in rural Iran. Lancet. 2012;379(9810):6–7.

44 Lehmann U, Van Damme W, Barten F, Sanders D. Task shifting: the answer to the human resources crisis in Africa? Hum Resour Health. 2009; 7:49.

46 Mdege ND, Chindove S, Ali S. The effectiveness and cost implications of task-shifting in the delivery of antiretroviral therapy to HIV-infected patients: a systematic review. Health Policy Plan. 2013; 28(3):223–36.

47 Lekoubou A, Awah P, Fezeu L, Sobngwi E, Kengne AP. Hypertension, diabetes mellitus, and task shifting in their management in sub-Saharan Africa. Int J Environ Res Public Health. 2010;7(2):353–63.

48 Gomez-Gonzalez JE, Reyes NR. Patterns of global health financing and potential future spending on health. Lancet. 2017;389(10083):1955–6.

49 Kankeu HT, Saksena P, Xu K, Evans DB. The financial burden from noncommunicable diseases in low- and middle-income countries: a literature review. Health Res Policy Syst. 2013;11:31.

51 De-Graft Aikins A. Healer shopping in Africa: new evidence from rural-urban qualitative study of Ghanaian diabetes experiences. BMJ. 2005; 331(7519):737.

52 Skirbekk V, Loichinger E, Weber D. Variation in cognitive functioning as a refined approach to comparing aging across countries. Proc Natl Acad Sci U S A. 2012;109(3): 770–4.

53 Bloom DE, Cafiero ET, Jané-Llopis E, Abrahams-Gessel S, Bloom LR, Fathima S, et al. The global economic burden of non-communicable diseases [Internet]. Geneva: World Economic Forum; 2011 Sep [cited 2017 Sep 26]. p. 29. Available from: http://apps.who.int/medicinedocs/documents/s18806en/s18806en.pdf

54 Abegunde DO, Mathers CD, Adam T, Ortegon M, Strong K. The burden and costs of chronic diseases in low-income and middle-income countries. Lancet. 2007;370(9603): 1929–38.

55 Skirbekk V, Ottersen T, Hamavid H, Sadat N, Dieleman JL. Vast majority of development assistance for health funds target those below age sixty. Health Aff (Millwood). 2017;36(5): 926–30.

56 Narayan KM, Miotti PG, Anand NP, Kline LM, Harmston C, Gulakowski R 3rd, et al. 3rd, HIV and noncommunicable disease comorbidities in the era of antiretroviral therapy: a vital agenda for research in low- and middle-income country settings. J Acquir Immune Defic Syndr. 2014; 67(Suppl 1):S2–7.

57 Janssens B, Van Damme W, Raleigh B, Gupta J, Khem S, Soy Ty K, et al. Offering integrated care for HIV/AIDS, diabetes and hypertension within chronic disease clinics in Cambodia. Bull World Health Organ. 2007;85(11):880–5.

59 Scholl E, Cothran D. Integrating family planning and HIV services: programs in Kenya and Ethiopia lead the way [Internet]. Arlington (VA): AIDSTAR-One; 2011 Mar [cited 2017 Sep 26]. Available from: https://aidsfree.usaid.gov/sites/default/files/integrating_family_planning_hiv_services.pdf

60 Bollyky TJ, Emanuel EJ, Goosby EP, Satcher D, Shalala DE, Thompson TG. NCDs and an outcome-based approach to global health. Lancet. 2014;384(9959):2003–4.

61 National Academies of Sciences, Engineering, and Medicine. Global health and the future role of the United States. Washington (DC): National Academies Press; 2017.

62 Vital Strategies [Internet]. New York: Vital Strategies. Press release, Vital Strategies launches Resolve, a new $225 million global health initiative; 2017 Sep 12 [cited 2017 Oct 6]. Available from: http://www.vitalstrategies.org/vital-stories/vital-strategies-launches-resolve-new-225-million-global-health-initiative/

